# *In Vivo* Measurement of Mesokinesis in *Gekko gecko*: The Role of Cranial Kinesis during Gape Display, Feeding and Biting

**DOI:** 10.1371/journal.pone.0134710

**Published:** 2015-07-31

**Authors:** Stéphane J. Montuelle, Susan H. Williams

**Affiliations:** Ohio University Heritage College of Osteopathic Medicine, Department of Biomedical Sciences, Irvine Hall 228, Athens, OH, 45701, United States of America; College of the Holy Cross, UNITED STATES

## Abstract

Cranial kinesis refers to movements of skeletal sub-units relative to one another at mobile sutures within the skull. The presence and functional significance of cranial kinesis has been investigated in various vertebrates, with much of our understanding coming from comparative studies and manipulation of ligamentous specimens. Drawing on these studies, cranial kinesis in lizards has been modeled as a four-bar linkage system involving streptostyly (rotation of the quadrate), hypokinesis (dorsoventral flexion and extension of the palato-maxillary sub-unit), mesokinesis (dorsoventral flexion and extension of the snout at the fronto-parietal suture) and metakinesis (sliding movements between parietal and supraocciptal bones). *In vivo* studies, although limited, suggest that cranial kinesis serves an important role during routine behaviors such as feeding. Here, we use X-ray Reconstruction Of Moving Morphology to further quantify mesokinesis *in vivo* in *Gekko gecko* during three routine behaviors: gape display, biting and post-ingestion feeding. During gape display, the snout rotates dorsally above rest position, with mesokinesis accounting for a 10% increase in maximum gape over that achieved solely by the depression of the lower jaw. During defensive biting, the snout rotates ventrally below rest position to participate in gape closure. Finally, ventroflexion of the snout also occurs during post-ingestion feeding, accounting for 42% of gape closure during intra-oral transport, 86% during puncture-crushing, and 61% during pharyngeal packing. Mesokinesis thus appears to facilitate prey puncturing by allowing the snout to rotate ventrally so that the upper teeth pierce the prey item, thus limiting the need for large movements of the lower jaw. This is suggested to maintain a firm grip on the prey and reduce the possibility of prey escape. More generally, this study demonstrates that mesokinesis is a key component of defensive biting and gape display behaviors, as well as post-ingestion feeding, all of which are linked to organismal fitness.

## Introduction

The skull is a complex anatomical system involved in a variety of behaviors linked to organismal fitness and the adaptive profile of individual species, such as communication with conspecifics, defense, and feeding. Consequently, cranial morphology reflects the different selective pressures associated with a wide range of behaviors. In many vertebrate lineages, the cranial bones have fused to protect the brain, but in some, the skull is kinetic, characterized by mobile joints within the cranium allowing movement of intracranial skeletal sub-units relative to one another. Cranial kinesis has been documented in osteichthyans [1–3], amphibians [4–5], lepidosaurs [6–13], and aves [14–17]. In lepidosaurs, cranial kinesis has been of particular interest in large part due to the highly derived and kinetic skulls of ophidian squamates (i.e., snakes) [11–13], but the form and function of cranial kinesis has also been studied in non-ophidians (i.e., *Sphenodon* and squamate lizards) (reviewed in [7–8]). At the morphological level, histological analyses reveal that cranial sutures in the squamate skull are more diverse than previously thought, suggesting different patterns of intra-cranial mobility [10, 18–20]. At the functional level, the skull of snakes has been shown to be very kinetic *in vivo* [11–13], but evidence is more scarce in lizards (but see [21–23]).

Previous research has identified 4 types of intra-cranial movements in lizards [6–10, 24]. Streptostyly is the rotation of the quadrate at its dorsal articulation with the squamosal and/or the supratemporal. Mesokinesis is the dorsoventral flexion and extension of the palato-maxillary unit (i.e., the snout) around an axis that runs transversely through the frontal-parietal suture. Hypokinesis is the dorsoventral flexion and extension of the palatine unit around an axis that runs transversely through the palate (palatine/ ectopterygoid/ pterygoid) suture, and is often considered complementary to mesokinesis. Finally, metakinesis is the curvilinear movements of the supraoccipital bones sliding anteroposteriorly under the parietal, suggesting changes in link length within the 4-bar linkage model. The presence/absence of each, some or all of these intra-cranial movements varies considerably across lizards taxa (reviewed in [7–8]). Nevertheless, cranial kinesis in lizards is typically modeled as a four-bar linkage in which, as the lower jaw depresses, the quadrate rotates rostrally at the streptostylic joint, pushing the palato-maxillary sub-unit forward and thus lifting the snout dorsally at the mesokinetic and hypokinetic joints [6–8]. As the lower jaw is adducted, the quadrate rotates caudally at its dorsal aspect around the streptostylic joint, pulling the palato-maxillary sub-unit posteriorly and thus depressing the snout ventrally at the mesokinetic and hypokinetic joints [6–8].

Inferences of cranial kinesis in lizards are mostly based on the manipulation of ligamentous specimens [7–8, 25] and have recently benefited from histological analyses of associated joints [10, 20] as well as computational simulations [18, 26–29]. However, movements at intra-cranial joints have been reported in some lizards *in vivo* [21–23, 30–32], but only during feeding, as the one hypothesis proposes that cranial kinesis in squamate lizards relates to prey prehension and manipulation capabilities [6–9]. In this hypothesis, cranial kinesis allows alignment of the upper and lower jaw during prey capture, which may be of particular relevance in lizards that use jaw prehension [6–7]. Movements at the mesokinetic joints in particular are hypothesized to allow “an instantaneous momentum reversal in the upper jaw as it changes from opening to closing without requiring head flexure at the neck” [7]. A second hypothesis is that cranial kinesis in geckoes is linked to skull bone reduction [33]. In this hypothesis, the loss of the postorbital and supratemporal bars and subsequent increase in cranial kinesis is associated with the increased eye size driven by their nocturnal lifestyle [25, 33]. Here, our goal is to expand our understanding of the function of mesokinesis by quantifying its contribution to other behaviors that are ecologically relevant: gape display, defensive biting, and post-ingestion feeding (i.e., prey processing and transport). We focus on *Gekko gecko* Linneus 1758, a species in which these behaviors have a direct link to survival and organismal fitness. Indeed, *G*. *gecko* individuals engage in gape display to establish and maintain territory against conspecifics and other threats [34]. When facing a threat or predator, *G*. *gecko* also uses bites as a defense mechanism [35]. Finally feeding is related to fitness as food is the source of nutrients and energy.

Cranial kinesis in *G*. *gecko* has been investigated previously during feeding using uniplanar fluoroscopy [21]. This study confirmed the 4-bar linkage model of cranial kinesis in *G*. *gecko* (and in another gekkonid species *P*. *madagascariensis*) with the mesokinetic joint of allowing for a 6° rotation of the snout relative to the braincase (dorsoflexion and ventroflexion) during processing of crickets [21]. Unfortunately, out-of-plane movements due to the animals tilting their head limited the dataset for which mesokinesis could be analyzed quantitatively [21]. Here, we use biplanar fluoroscopy and X-ray Reconstructions Of Moving Morphology (XROMM) [36] as it allows reconstructions of bone movements in 3 dimensions regardless of the positioning of the subject in the field of view.

We focus on mesokinesis because hypotheses based on the classical model for cranial kinesis suggest that it may be important for gape and bite force production, as well as for feeding [6–9, 33]. When signaling against predators, elevation of the snout is hypothesized to contribute to gape opening (here defined as the linear dorsoventral distance of the oral opening). Gape opening results primarily from jaw depression, but is expected to be amplified by the elevation of the snout at the mesokinetic joint. In order to evaluate the contribution of mesokinesis to gape display, we compare gape distances measured *in vivo* (i.e., with mesokinesis movements) to a ‘theoretical akinetic gape’ simulated with no movement at the mesokinetic joint. In contrast, ventral rotation of the snout during jaw closing is hypothesized to be correlated with (i) bite force during defensive biting and (ii) gape closing distance during post-ingestion feeding behaviors. These hypotheses are based on previous modelling of cranial kinesis in geckoes [25] that found that “bite force increases by up to 15% in *G*. *gecko* as the result of the retraction of the kinetic system” [25]. These hypotheses are investigated by testing the correlation between ventral rotation of the snout at the mesokinetic joint and (i) the magnitude of bite force and (ii) the amplitude of gape closing movements. Defensive biting is a relatively straightforward behavior that has been a hallmark of performance studies in lizards and other vertebrates, and has been demonstrated to be advantageous as means of defense [35]. Moreover, biting in lizards also increases feeding performance by reducing the number of bites necessary to process food item [37–38]. Feeding, on the other hand, involves different types of gape cycles which may be associated with differences in the magnitude of mesokinetic movements. In accordance with the four-bar linkage model [6–8], an increase in the amplitude of gape closing is expected to be correlated with an increase in absolute movements at the mesokinetic joint. Whereas transport cycles are expected to be characterized by large gape closing movements (i.e., amount of jaw closing starting from a large gape) to accommodate prey repositioning within the oral cavity, other cycles like puncture-crushing dedicated to altering the integrity of the prey structure are expected to be characterized by small gape closing movements (i.e., amount of jaw closing starting from a small gape). Accordingly, absolute amplitude of mesokinesis during transport cycles is expected to be greater than during other types of cycles.

Because the amplitude of jaw movements varies during feeding in lizards (i.e., between cycles corresponding to different feeding behaviors) [7, 39–41], we also scale mesokinetic movements to the amplitude of gape closing to determine whether there are differences among intraoral transport, processing and pharyngeal packing cycles independent of differences in the amplitude of gape closing. Scaled mesokinetic movements are expected to show that the contribution of ventral rotation of the snout at the mesokinetic joint is greater during feeding cycles characterized by small gape closing movements. Previously, mesokinetic movements have indeed been hypothesized to help “maintain a secure grip on the prey” [31], although this was based on qualitative observations. Here puncture-crushing cycles are hypothesized to be characterized by (i) small absolute mesokinetic movements, but (ii) large scaled mesokinetic movements, highlighting the importance of mesokinesis for bite force production at small gapes. In contrast, feeding behaviors characterized by wide gape closing movements, such as intra-oral transport cycles, are hypothesized to be characterized by (i) large absolute mesokinetic movements, but (ii) small scaled mesokinetic movements as adduction of the mandible is expected to be the primary contributor to jaw closing. Indeed, in this latter case, the contribution of mesokinesis to gape closing may be overshadowed by the amplitude of jaw adduction.

## Material and Methods

### Husbandry

Five male individuals were obtained from local breeders (snout-vent length: 123.9 mm ± 3.5; skull length: 39.9 mm ± 2.0; body weight: 33.7 g ± 1.7; obtained commercially at Fish N’ Stuff, Athens, OH, 45701, USA). All individuals were housed individually in 30 cm x 30 cm x 45 cm terrarium in a room with a 12:12 day-night light cycle and 70% humidity. Terraria were placed next to one another with opaque paper sheets in between to prevent stress. Water was made available ad-libitum and 4 to 5 crickets were offered to each individual daily. However, food was withheld for 2 days prior to recording sessions to facilitate the collection of feeding data. After sufficient data collection, individuals were euthanized via intraperitoneal injection of a commercial veterinary euthanasia solution.

### X-ray Reconstruction Of Moving Morphology XROMM

#### 1. Marker implantation and construction of 3D bone models

**Fig 1:**
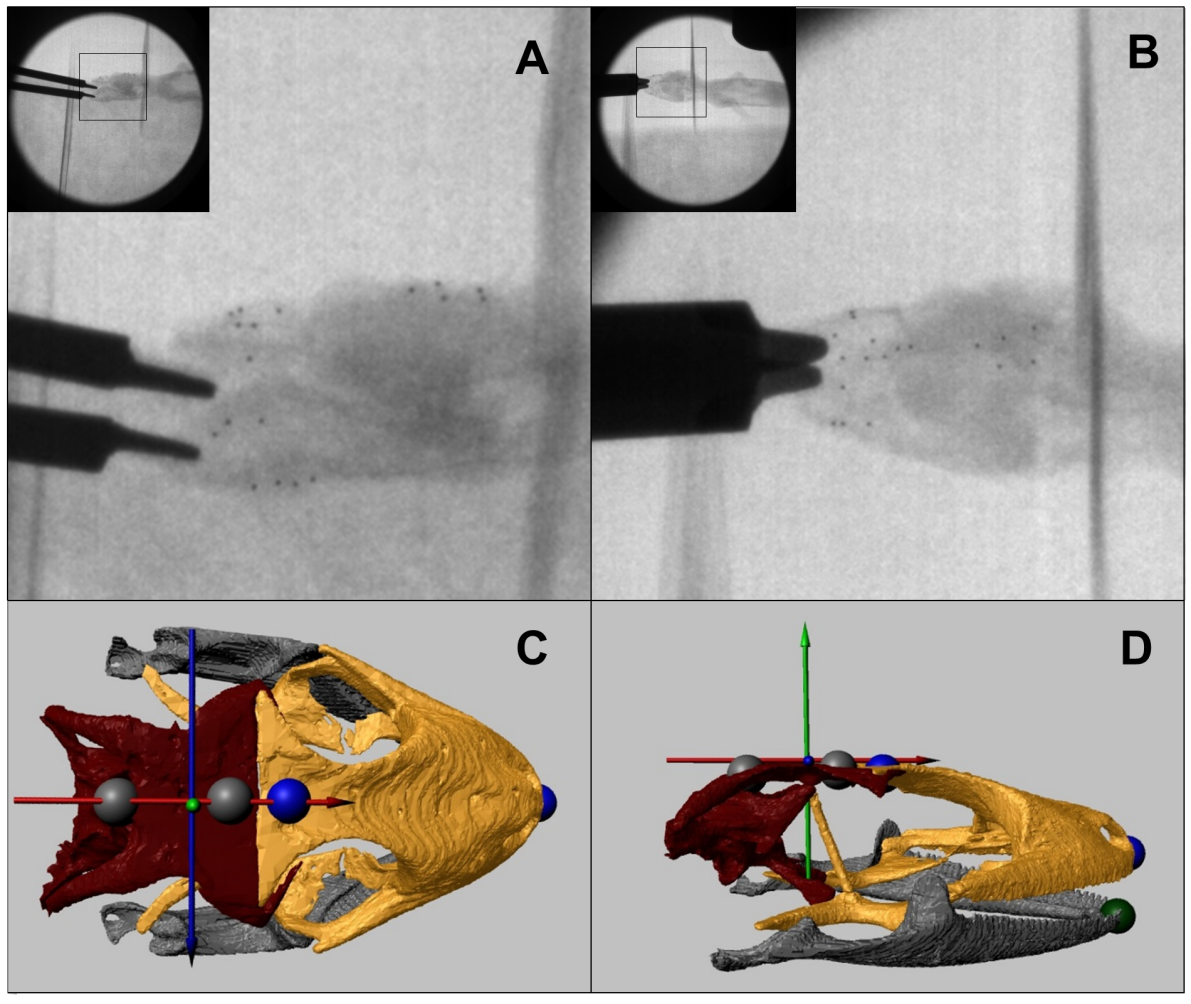
A-B. Representative biplanar fluoroscopy frames of *Gekko gecko* biting on a bite force transducer (recorded at 250 frames per seconds). In each view, the screen coordinates of the implanted radiopaque markers (6 in the snout, 5 in the braincase, 3 in the right lower jaw and 4 in the left lower jaw) are digitized throughout the sequence to calculate the 3D position of the elements of interest (the snout, the braincase and the lower jaw). The snout of the subject is oriented to the right. **C-D. Still frames extracted from an XROMM animation of *Gekko gecko* biting**. Mesokinetic angle is calculated as the angle between the anterior-posterior axis of the snout (in gold, defined by 2 landmarks in blue) and the anterior-posterior axis of the braincase (in red, defined by 2 landmarks in grey). A fifth landmark (green) was defined at the tip of the lower jaw to measure gape distance. Note that these are virtual landmarks created in the XROMM animations; i.e., they are different than the markers surgically implanted. XROMM animations allow quantifying the movements of the snout and of the lower jaw relative to the braincase reference system: antero-posterior axis (red axis), dorso-ventral axis (green axis) and medio-lateral axis (blue axis). See S1 File for an example of full XROMM animation.

Surgeries to implant radiopaque markers for subsequent fluoroscopy and XROMM were performed on one subject at a time. Anesthesia was induced with an intramuscular injection of 40 mg/kg of ketamine mixed with 200μg/kg of dexmedetomidine, following published protocols [42]. First, an incision was made through the scales overlaying the target bone. Because of the size of the bones, one incision was usually sufficient to implant 3 or more markers. After soft tissues were delicately pushed aside to expose a suitable implantation site, a sterilized stainless micro drill was used to drill a hole in the target bone. A 0.5-mm-diameter radiopaque tantalum bead (Bal-tec, Los Angeles, CA, USA) was then inserted in the hole and secured with cyanoacrylate adhesive. Five markers were implanted in the parietal bones, 6 in the snout (frontal, nasal and maxilla bones), and 7 or 8 markers in the left and right dentaries (i.e., 8 total in the jaw) (Fig 1A and 1B). Beads were implanted as far apart as possible from one another in the bone of interest to reconstruct rigid body motion as accurately as possible. Note that, despite anatomical evidence suggesting mobility at the mandibular symphysis [19], the mandible was considered a single unit, and mobility at the mandibular symphysis was not investigated in the present study.

While still under anesthesia, the subject was scanned using a GE eXplore Locus in vivo Small Animal MicroCT Scanner (90 micron slices, 80 kVp, 450 μA) to create 3D bone models in Avizo (FEI Visualization Sciences Group, Burlington, MA, USA). After the scan was complete, 200μg/kg of atipamezole were injected to reverse the effects of dexmedetomidine [42–43]. A 48-hour long recovery period was strictly observed before starting the experiments to make sure the subject was behaving naturally after the surgery.

#### 2. Distortion correction and calibration

Two synchronized high-speed cameras (Oqus 310, Qualisys, Sweden) were mounted on the output ports of two fluoroscopes (OEC-9000). High-speed video cameras and a Logitech webcam C210 were synchronized in the Qualisys Track manager software. The webcam was used to provide an external view of the animal during fluoroscopy recordings. In order to correct for distortion inherent to x-ray imaging, a perforated steel sheet with standardized hole spacing and sizes (part number 9255T641, McMaster-Carr, Robinson, NJ; i.e., distortion-correction grid) was imaged in each fluoroscopy view [36]. A correction algorithm in Matlab undistorts the images of the distortion-correction grid, creating an undistortion matrix that is then applied to all subsequent fluoroscopy images.

The field of view covered by both fluoroscopes was calibrated by exposing a custom cube of 4 plastic sheets containing 16 radiopaque tantalum beads placed in a 4 x 4 fashion 2.5 cm apart from one another. A 3D model based on a micro-CT scan of the cube provided the reference x, y and z coordinates of each of the 64 beads. The calibration cube was imaged with both fluoroscopes and the screen position of each bead was digitized on each view. Following the XROMM workflow [36, 44], direct linear transformation (DLT) was calculated to obtain the 3D position of each video camera. Mean DLT residuals across recording sessions was 0.409 mm ± 0.02 s.e.m. The distortion-correction grid and the calibration cube were recorded at the beginning and end of each recording session to prevent the loss of data due to any accidental changes in the position of the fluoroscopes over the course of a session.

#### 3. Fluoroscopy recordings and bite force measurement

Fluoroscopy movies and bite force measurements were recorded daily within a two-week time period. All video recordings were set at 250 frames per second. Specific details on the recording sessions of each behavior (gape display, biting, feeding) are provided below.

As the mere handling of the subjects immediately triggered defensive gaping, sequences of gape displays were recorded while holding the subjects in the field of view of the fluoroscopes. Multiple 1-second long video sequences of defensive gaping were recorded. Although subjects became accustomed to being handled within a single recording session, thus eliciting submaximal gapes, alternating between hands every other trial maintained the level of stimulus between trials. Recording sessions were stopped when the subject required additional stimulation (i.e., gently tapping on the snout) to perform a gape display. The experimenter holding the animal wore the full complement of radiation safety clothing, including lead-lined gloves.

Bite force measurements were made using a strain gauge-based bite force transducer (designed after [45]) connected to a Vishay Micro-Measurements 2120B strain conditioner (similarly to [46]). Voltage output from the conditioner was recorded at 500 Hz and synchronized with the high-speed cameras. Subjects were placed in a radiolucent box to control the position of the subject within the field of view. The box had an opening for the neck and the head. The bite force transducer was presented in front of the subject to trigger spontaneous biting behavior, during which time fluoroscopy movies and voltage output were recorded simultaneously. The number of bite replicates within each sequence was variable. Recording sessions ended when the subject refused to bite the transducer. At the end of each recording session, the bite force transducer was calibrated using weights between 1kg and 500g in 100-g increments. The peak voltage output for each weight was regressed against its calibration weight. For each recording session, the relationship between the weight and the voltage output was linear and repeatable, and over the course of the whole experiment, the calibration of the bite force transducer was also repeatable (mean R^2^ = 0.9832; ranging from 0.9576 and 0.9965).

The diet of *Gekko gecko* in the wild has been reported to be mainly insectivorous [47]. Therefore, to collect feeding data, live crickets (*Acheta domesticus* 11.2 mm ± 0.4) were offered to each subject. The subjects were placed in the same box used for the bite force recording session and a cricket was placed in their mouth as, across all recording sessions, the subjects refused to engage in prey capture voluntarily. Feeding sequences were recorded as soon as the subject spontaneously started to bite, process and transport the food item through the oral cavity. Following the nomenclature of feeding behaviors described previously [7], gape cycles were identified as intra-oral transport, puncture-crushing or pharyngeal packing. Intra-oral transport cycles were considered the gape cycles during which the prey was visible between the tooth rows at the beginning of the cycle and transported posteriorly within the intra-oral cavity during that cycle. Puncture-crushing cycles were the gape cycles during which the jaws open and close onto the prey item to alter its physical and textural integrity without reducing it into smaller pieces [7]. Note that no intra-oral transport was observed during puncture-crushing cycles, i.e., the prey is stationery in the oral cavity (in accordance with [48]), and no distinction was made here as to the mechanics of prey alteration (puncture, crush or both; see [7]) because this was difficult to determine from the movies. Finally, pharyngeal packing cycles were the gape cycles during which the prey was pushed into the esophagus and swallowed. These cycles involved opening-closing of the jaws while the tongue protruded outside of the oral cavity and retracted rapidly to push the prey item further posteriorly through the pharynx towards the oesophagus. Cycle types were identified according to the position of the prey within the oropharyngeal tract using the light camera and fluoroscopy footage.

#### 4. Digitization, animation and anatomical coordinate system

The screen position of each implanted marker was digitized in each view of each sequence following the XROMM workflow [36]. Using the DLT calibration, the 3D coordinates of each marker were calculated using the MatLab-based XROMM modules [36, 44]. These were then used to calculate the rigid body motion of the three cranial sub-units of interest (i.e., the braincase, the snout and the lower jaw) using singular value decomposition [35]. Rigid body motions were applied to the corresponding 3D models in Maya (S1 File), and the frame-by-frame animation of each recorded sequence allowed visualizing and quantifying bone movements. Over the entire data set, the average standard deviation of the distance between markers implanted in the same bone was 0.144 mm ± 0.0010.

An anatomical reference system consisting of 3 axes perpendicular to each other was set with the origin point (coordinates 0, 0, 0) at the intersection of the frontal-parietal suture with the interparietal suture (bregma). The x-axis was defined as the antero-posterior axis (AP; i.e., parallel to the interparietal suture line, pointing towards the snout), the y-axis as the medio-lateral axis (i.e., defined by the frontal-parietal suture) and the z-axis as the dorsoventral axis (Fig 1C and 1D). This reference system was parented to the parietal bones so that all movement was measured independently of head movements. To understand mobility at the mesokinetic joint, two points were identified on the dorsal surface of the snout model to create the AP axis of the snout. These points were located at the tip of the snout and at the posterior end of the midline of the frontal bone. The frontal bone is not paired in gekkotan lizards [20, 49] and thus lacks a suture line to define its midline. Instead, the midline of the frontal bone was approximated by extending the interparietal suture line. Similarly, two points were defined on the dorsal surface of the braincase model to create the AP axis of the braincase. These points were located at the anterior and posterior ends, respectively, of the parietal suture line. All 4 points were located in the mid-sagittal plane.

For each individual, resting mesokinetic angle was calculated as the angle created by the intersection of the AP axis of the snout and the AP axis of the braincase from the 3D reconstruction of the micro-CT scan using the AP braincase and snout axes. The microCT scans were used because the animals were completely anesthetized with their jaws fully closed and showing no muscular tone. The resting mesokinetic angle was used as a baseline to evaluate the extent of dorsiflexion and/or ventroflexion of the snout that occurs *in vivo* during the different behaviors investigated relative to a neutral or passive position. Note mesokinetic angle at rest position varied among subjects: 154.8°, 157.6°, 147.2°, 154.0°, and 165.6° for each subject, respectively.

#### 5. Kinematic variables

For each XROMM animation, 3D coordinate data were used to calculate 8 kinematic variables (Table 1). The mesokinetic angle was calculated frame-by-frame throughout the sequence as the angle between the AP axis of the snout and the AP axis of the braincase. Mesokinetic displacement is the difference between the mesokinetic angle measured in the XROMM animation and the resting mesokinetic angle (taken from the 3D reconstructions; see above). Positive mesokinetic displacement values indicate dorsiflexion of the snout above resting position, and negative values represent ventroflexion of the snout below resting position. Maximum mesokinetic displacement (thereafter, maximum dorsiflexion of the snout) was extracted for each gape display and feeding cycle. Minimum mesokinetic displacement (thereafter, maximum ventroflexion of the snout) was determined for each bite and feeding cycle. For each feeding cycle, we also calculated the total mesokinetic movement and the relative mesokinetic movement. Total mesokinetic movement is the difference between maximum dorsiflexion and maximum ventroflexion of the snout. Finally, relative mesokinetic movement was calculated as the mesokinetic movement scaled to the associated gape closing distance because the amplitude of gape closing was different across the post-ingestion feeding behaviors under investigation.

**Table 1:** Variables and terminology used in this study.

Variables	Calculation and / or description
Mesokinetic angle	Angle between AP axis of the snout and AP axis of the braincase; calculated for each frame throughout the XROMM animations
Mesokinetic displacement	Difference between Mesokinetic angle and Resting mesokinetic angle; Maximum mesokinetic displacement = maximum dorsiflexion of the snout; Minimum mesokinetic displacement = maximum ventroflexion of the snout
Total mesokinetic movement	Difference between Maximum dorsiflexion and Maximum ventroflexion of the snout within a single feeding cycle; calculated only for feeding cycles
Relative mesokinetic movement	Absolute mesokinetic movement / jaw closing distance; calculated only for feeding cycles
Gape	Distance between the anteriormost point on the upper jaw and the anteriormost point on the lower jaw
Gape closing distance	Difference between Maximum gape and Minimum gape; calculated only for feeding cycles
Akinetic gape distance	Distance between the anteriormost point on the lower jaw and the anteriormost point on the upper jaw as if the skull were akinetic (i.e., fused frontal-parietal suture); calculated for gape display and feeding cycles
Contribution of mesokinesis movement to gape distance	Difference between Gape distance measured *in vivo* (i.e., with mesokinesis) and Akinetic gape distance (i.e., calculated as if the skull were akinetic); expressed in % of akinetic gape distance; calculated for gape display and feeding cycles only

For each frame in the feeding and gape display sequences, we also computed gape distance as the distance between the x-y-z coordinates of the tip of the lower jaw and the x-y-z coordinates of the tip of the upper jaw. An akinetic gape distance profile was also computed as if the frontal-parietal suture were fused in rest position. In this case, the snout and parietals were considered as a single model animated from the movements of the parietals, and gape was calculated as above. Thus the akinetic gape profile is solely based on depression of the lower jaw but includes any cranial elevation that occurred during the behavior recorded. For each gape display sequence recorded, the akinetic maximum gape distance was extracted at the instant of maximum depression of the lower jaw in each sequence. Maximum gape distance measured *in vivo* was then converted to a % of the akinetic value to quantify the extent to which mesokinesis contributes to gape during gape display. Similarly, for each feeding sequence, the akinetic gape closing distance was calculated, and the gape closing distance measured *in vivo* was converted to a % of the akinetic value to quantify the extent to which mesokinesis contributes to gape closing during post-ingestion feeding behaviors.

### Data analysis

**Fig 2:**
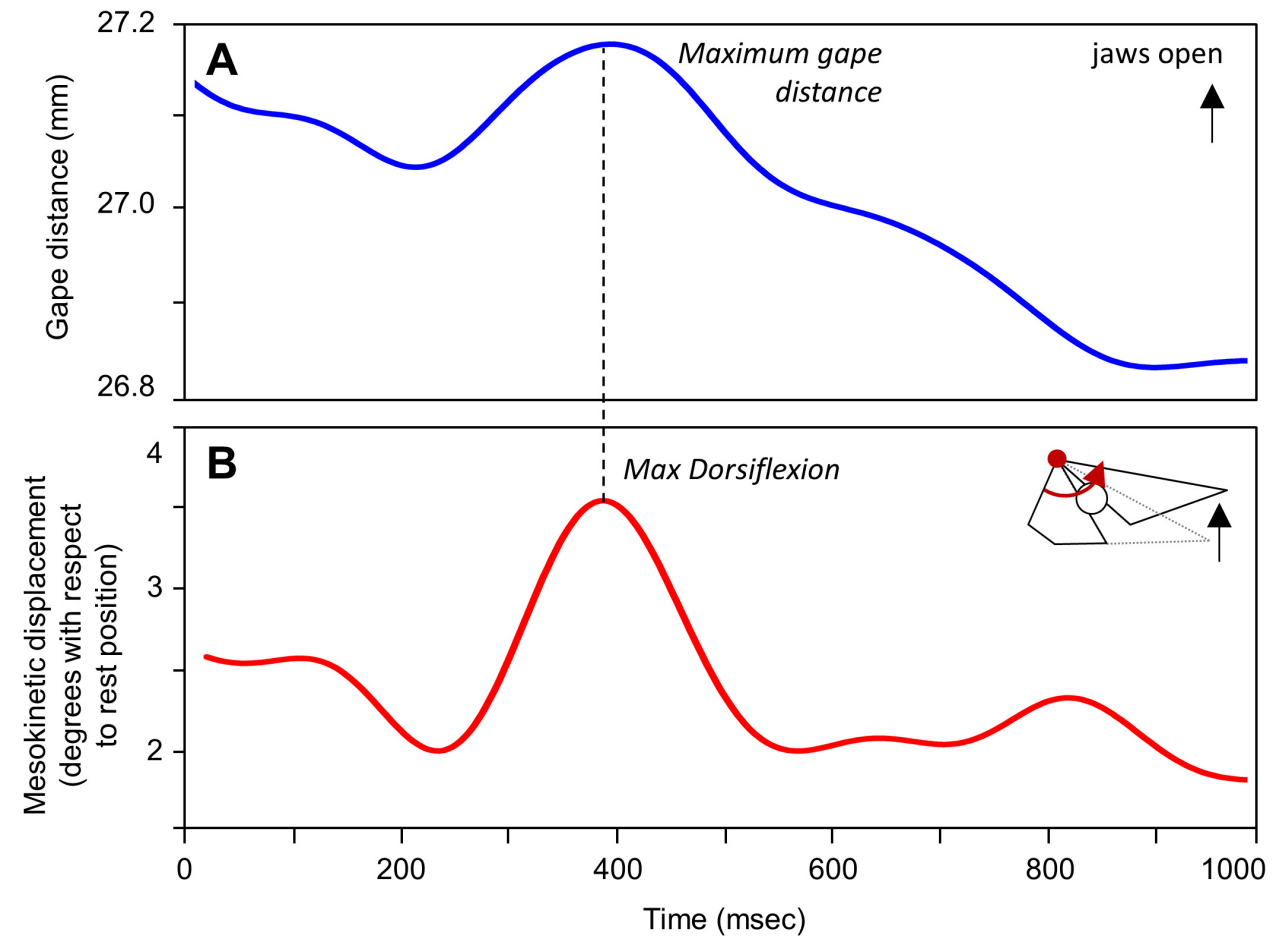
Mesokinesis during gape display in *Gekko gecko*. Representative traces show **(A)** gape (i.e., the distance between the tip of the upper and lower jaw) and **(B)** the associated mesokinetic displacement over time. Note that the snout flexes dorsally above rest position during gape display, and maximum gape occurs simultaneously with maximum dorsal flexion of the snout relative to the braincase.

To understand the role of mesokinesis during gape display, maximum gape was identified in each gape display sequence, along with the corresponding (i.e., simultaneous) mesokinetic angle (Fig 2). The associated mesokinetic displacement was then calculated. Bivariate correlations between maximum gape and mesokinetic displacement were tested with all individuals pooled together and for each individual separately. Individual differences in maximum gape and mesokinetic displacement were controlled using a multiple analysis of variance (MANOVA) with the individual factor entered as the random factor.

**Fig 3:**
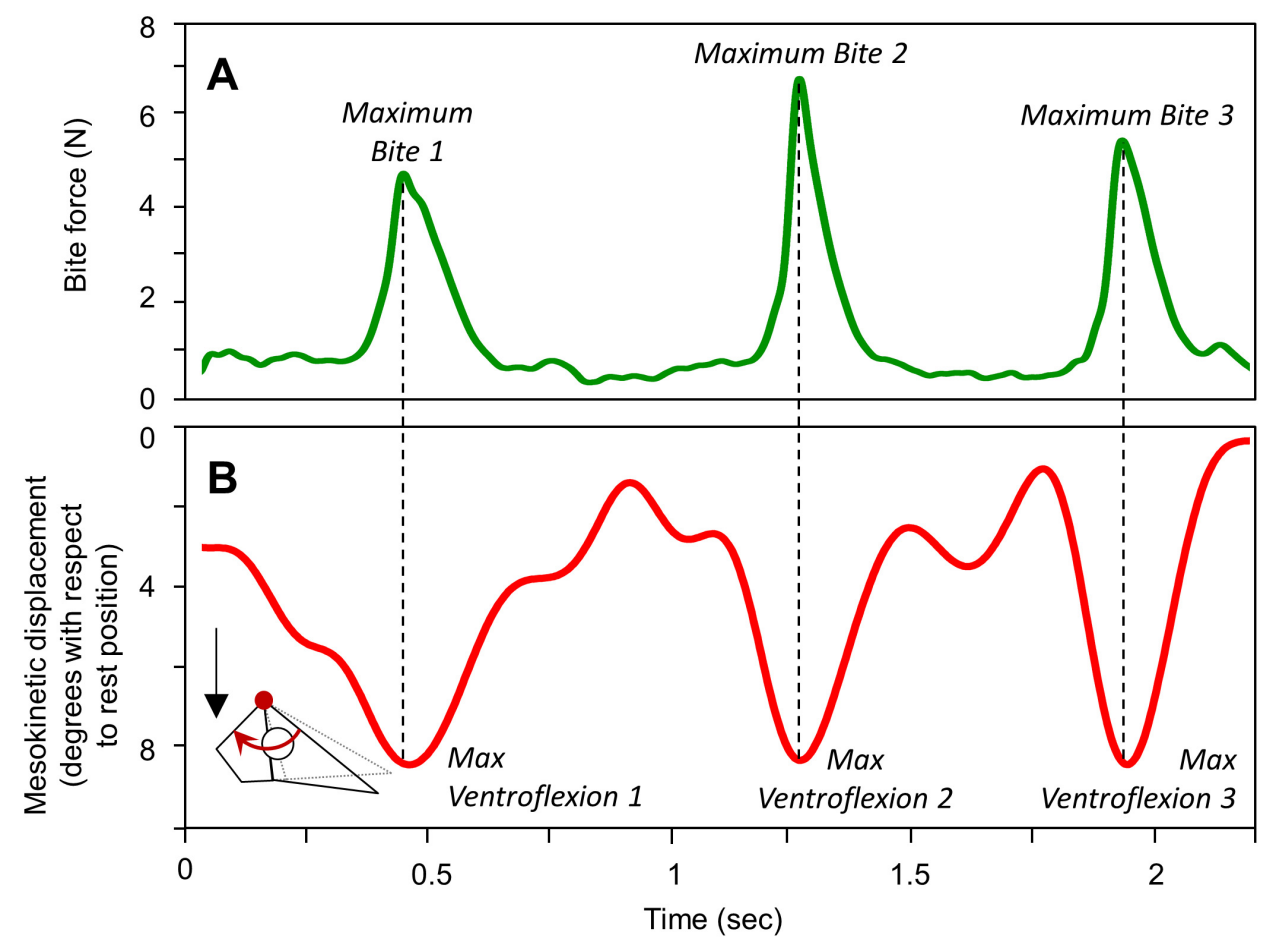
Mesokinesis during defensive biting in *Gekko gecko*. Representative traces show **(A)** bite force measured with a bite force transducer and **(B)** the associated mesokinetic displacement over time. In the sequence presented, 3 bites were recorded measuring 4.79 N, 6.84 N and 5.50 N, respectively. Note that the snout flexes ventrally below rest position during biting, and maximum bite force occurs simultaneously with maximum ventroflexion of the snout relative to the braincase.

To understand the role of mesokinesis during defensive biting, bite force replicates were extracted for each of the bites recorded within each sequence (Fig 3A). The corresponding (i.e., simultaneous) mesokinetic angle was extracted to calculate the mesokinetic displacement (Fig 3B). Bivariate correlations between bite force and mesokinetic displacement were tested with all individuals pooled together and for each individual separately. Individual differences in bite force and mesokinetic displacement were controlled using a MANOVA with the individual factor entered as the random factor.

**Fig 4:**
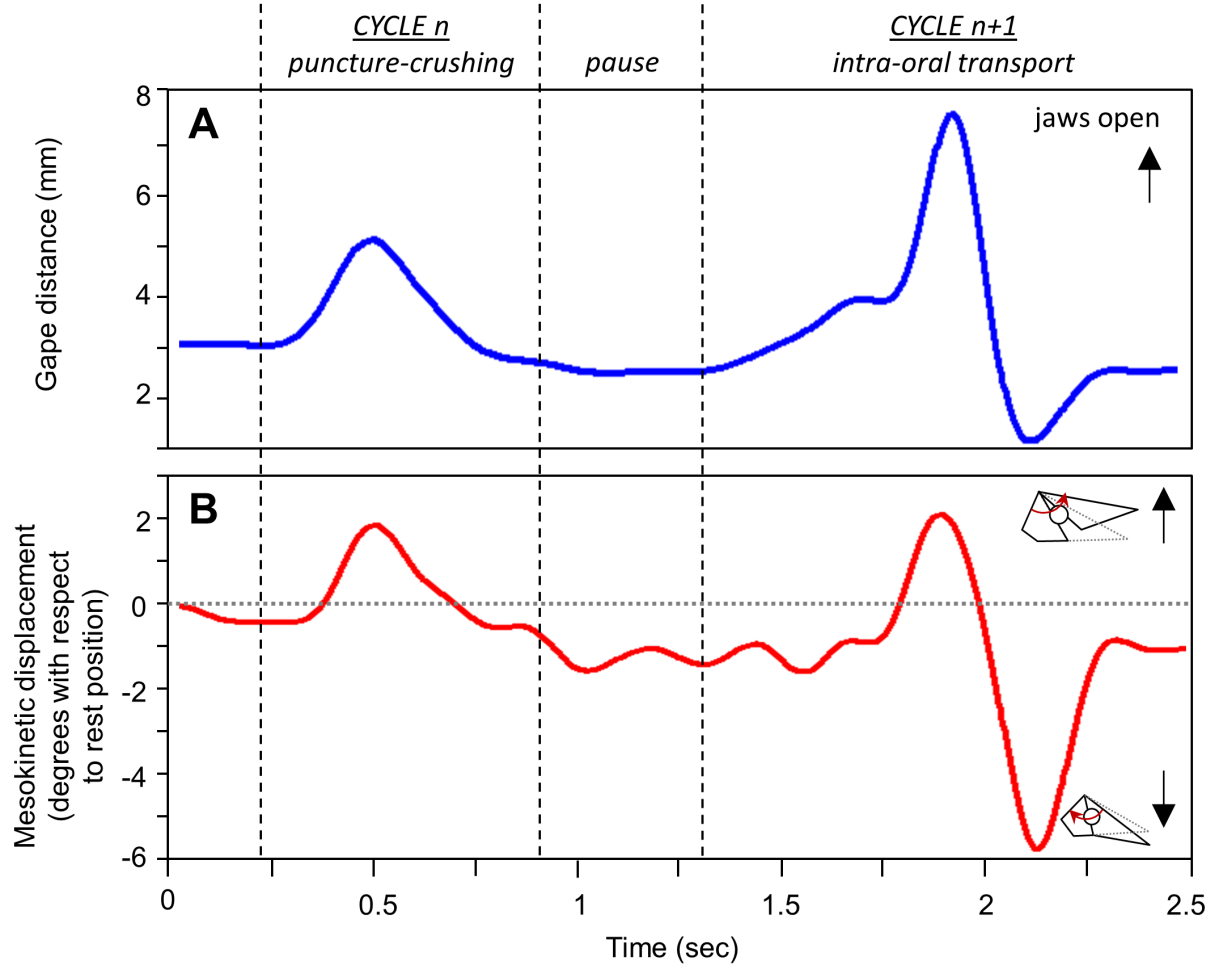
Mesokinesis during intra-oral transport and puncture crushing cycles in *Gekko gecko*. Representative traces show (A) gape and (B) the associated mesokinetic displacement over time. The horizontal dotted line represents the mesokinetic angle at rest position in the individual represented.

**Fig 5:**
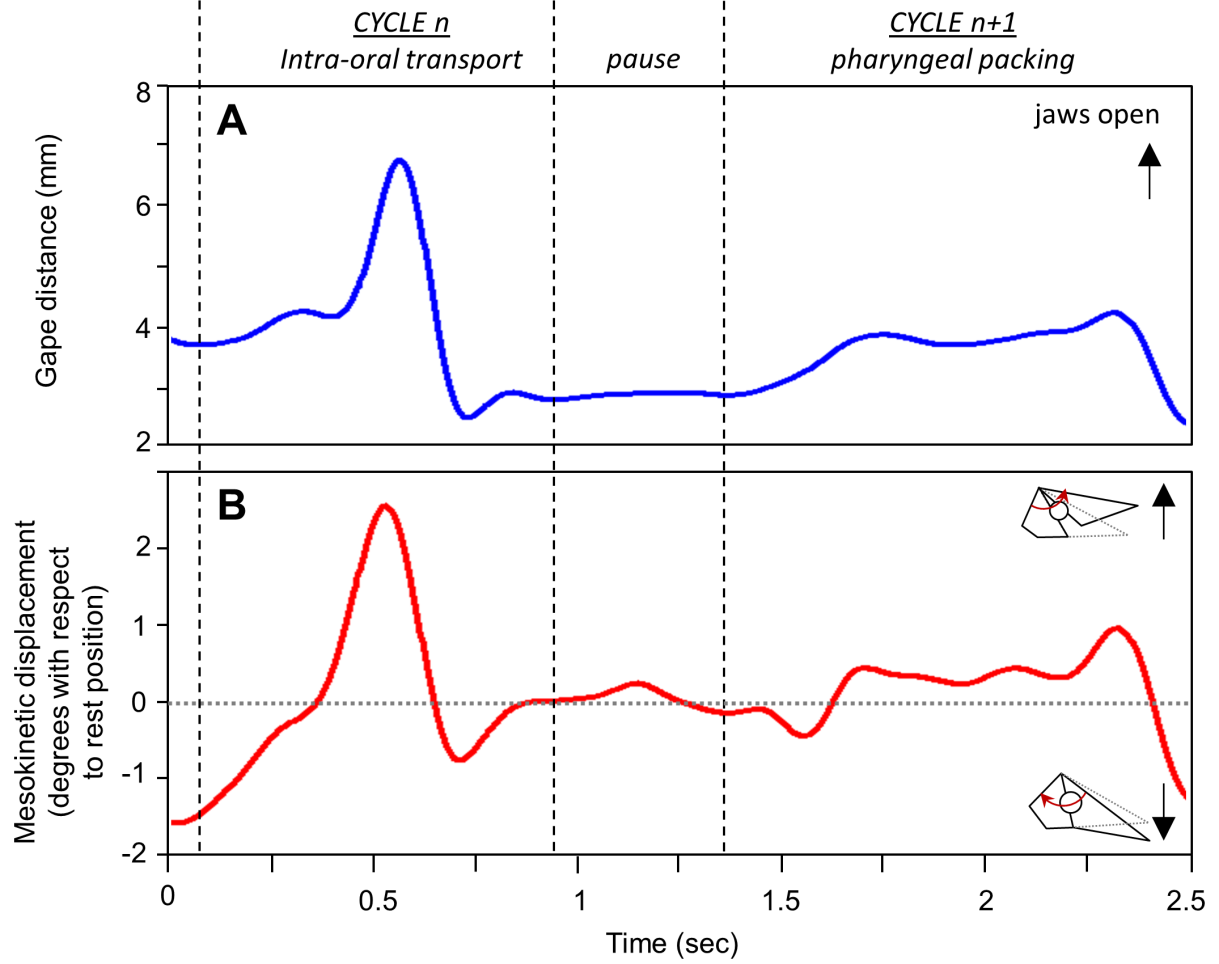
Mesokinesis during intra-oral transport and pharyngeal packing cycles in *Gekko gecko*. Representative traces show (A) gape and (B) the associated mesokinetic displacement over time. The horizontal dotted line represents the mesokinetic angle at rest position in the individual represented.

To understand the role of mesokinesis during post-ingestion feeding, gape closing distance and the associated mesokinetic movement were extracted for each cycle (Figs 4 and 5). Separate analyses of variance (ANOVAs) coupled to univariate *F*-tests were performed on the absolute and scaled mesokinetic movements with cycle type (intra-oral transport, puncture crushing, pharyngeal packing) entered as a fixed factor, individuals as a random factor, and the individuals x cycle type interaction term. A separate ANOVA of identical design was also performed on the amplitude of gape closing distance to test for differences in jaw movements among feeding cycle types. Finally, an ANOVA was performed on the contribution of mesokinesis to gape closing distance to test differences among cycle types (fixed factor), and individuals (random factor), as well as the cycle x individual interaction term. Bonferroni *post hoc* tests were used to test differences between cycle types. For all the analysis, the sample size for each individual is presented in Table 2 and the entire data set is attached in S2 File. All statistical procedures were performed using SPSS 13.0 (IBM, USA).

**Table 2:** Summary of the study sample. The number of replicates is presented for the complete data set and for each of the five individuals.

	Gape display	Defensive bites	Intra-oral transport	Puncture Crushing	Pharyngeal packing
All data	342	242	36	20	32
Individual 1	70	49	20	13	20
Individual 2	56	43	-	-	-
Individual 3	50	57	-	-	-
Individual 4	106	59	10	4	9
Individual 5	60	34	6	3	3

## Results

### Gape display

**Fig 6:**
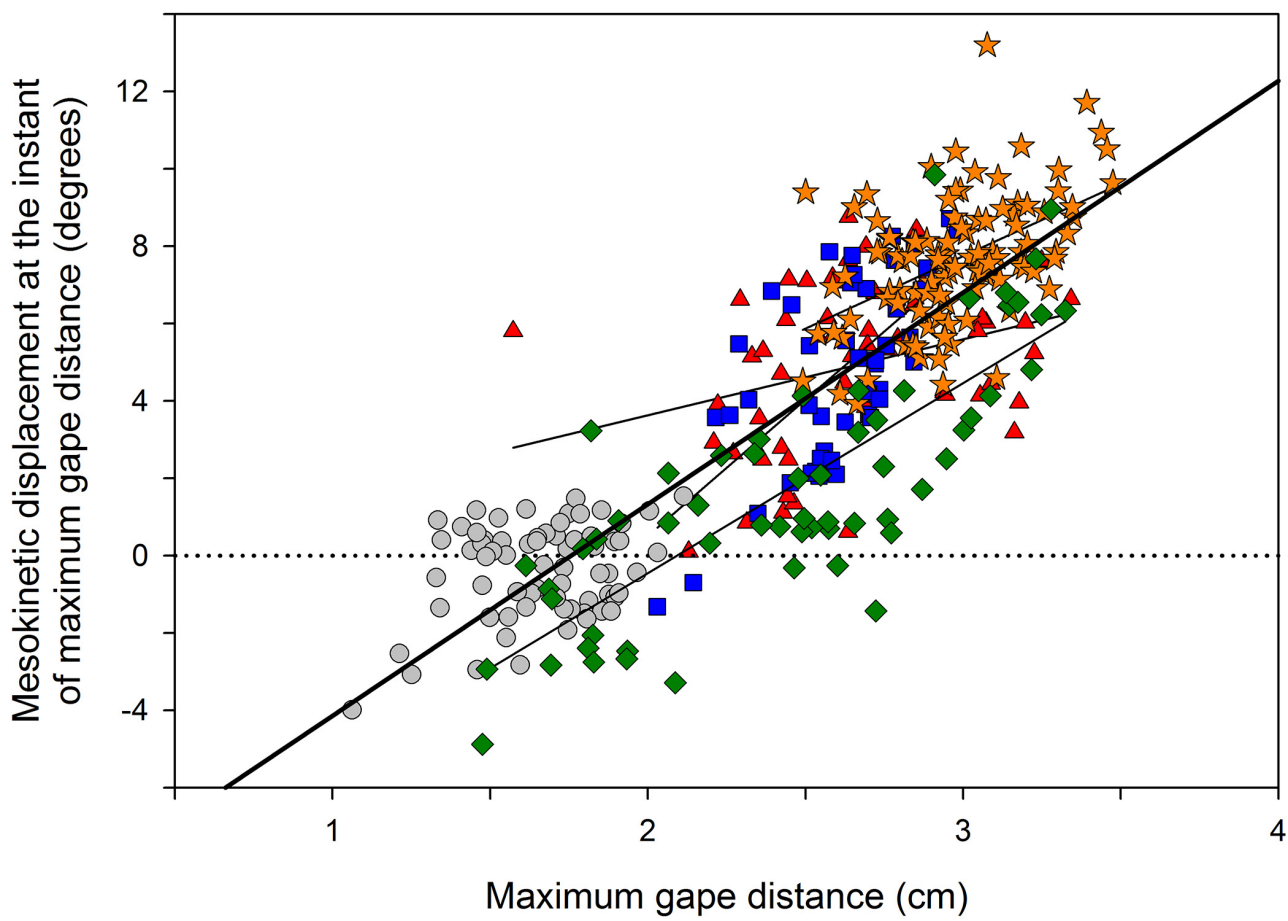
Correlation between maximum gape and dorsal rotation of the snout at the mesokinetic angle at maximum gape during gape display in *Gekko gecko*. The correlation is significant across all sequences recorded in this study (bold line), as well as at the individual level for 4 of the 5 individuals studied (continuous lines; see Table 4 for correlation parameters). Symbols represent individuals. Dotted line represents rest position: positive values indicate dorsal rotation of the snout above rest position; negative values indicate ventral rotation of the snout below rest position.

Fig 2 presents the kinematic profiles of gape distance (Fig 2A) and mesokinetic displacement (Fig 2B) through a representative 1-second long sequence of gape display behavior. In this sequence, the subject displays a maximum gape distance of 27.19 mm which is associated with 3.5° dorsiflexion of the snout at the mesokinetic joint. On average, the snout extends dorsally 4.10° (± 0.20 s.e.m.) above its neutral position during gape display (Table 3). Across individuals, maximum gape is correlated with mesokinetic displacements (*r* = 0.802, *P* < 0.001; Fig 6; Table 4), indicating that the greater the dorsal rotation of the snout relative to the braincase at the mesokinetic joint, the greater the maximum gape. This correlation was observed at the individual level in four of the five individuals studied (Fig 6; Table 4).

**Table 3:** Summary of mesokinetic displacements (mean ± standard error of the mean) observed in *Gekko gecko* during gape display and defensive biting.

Behavior	N	Mesokinetic displacement (degrees)^1^	Performance measure
Gape display	342	4.10° ± 0.20	*Maximum gape distance* 25.02 ± 0.30 mm
Defensive bite	242	-10.99° ± 0.44	*Maximum bite force* 11.38 ± 0.32 N

^1^ Positive values indicate dorsal rotation of the snout above rest position; negative values indicate ventral rotation of the snout below rest position.

**Table 4:** Summary of the correlations between mesokinetic displacements and performance in *Gekko gecko* during gape display and defensive biting.

	Gape display	Defensive bites
All data	*N* = 342, *r* = 0.802, *P* < 0.001	*N* = 242, *r* = -0.115, *P* = 0.07 (NS)
Individual 1	*N* = 70, *r* = -0.082, *P* = 0.501(NS)	*N* = 49, *r* = -0.545, *P* < 0.001
Individual 2	*N* = 56, *r* = 0.314, *P* = 0.019	*N* = 43; *r* = -0.641, *P* < 0.001
Individual 3	*N* = 50, *r* = 0.654, *P* < 0.001	*N* = 57, *r* = -0.446, *P* = 0.001
Individual 4	*N* = 106, *r* = 0.496, *P* < 0.001	*N* = 59, *r* = -0.633, *P* < 0.001
Individual 5	*N* = 60, *r* = 0.771, *P* < 0.001	*N* = 34, *r* = -0.144, *P* = 0.416 (NS)

Table entries are number of sequences analyzed (*N*), the Pearson correlation coefficient (*r*) and the significance level (*P*; NS indicates non-significant correlation).

The contribution of dorsoflexion of the snout at the mesokinetic joint to maximum gape was investigated by simulating gape as if the snout was at rest position in each of the 342 gape display sequences recorded. Simulations indicate that, on average, the maximum gape of an akinetic skull is 22.80 mm (± 0.03 s.e.m) which represents 91.3% of the maximum gape produced by a skull with mobility at the mesokinetic joint (2.50 ± 0.03 mm). This demonstrates that mobility at the frontal-parietal suture allows the snout to rotate dorsally above its rest position with respect to the braincase to produce an 8.7% increase in maximum gape.

### Defensive biting

**Fig 7:**
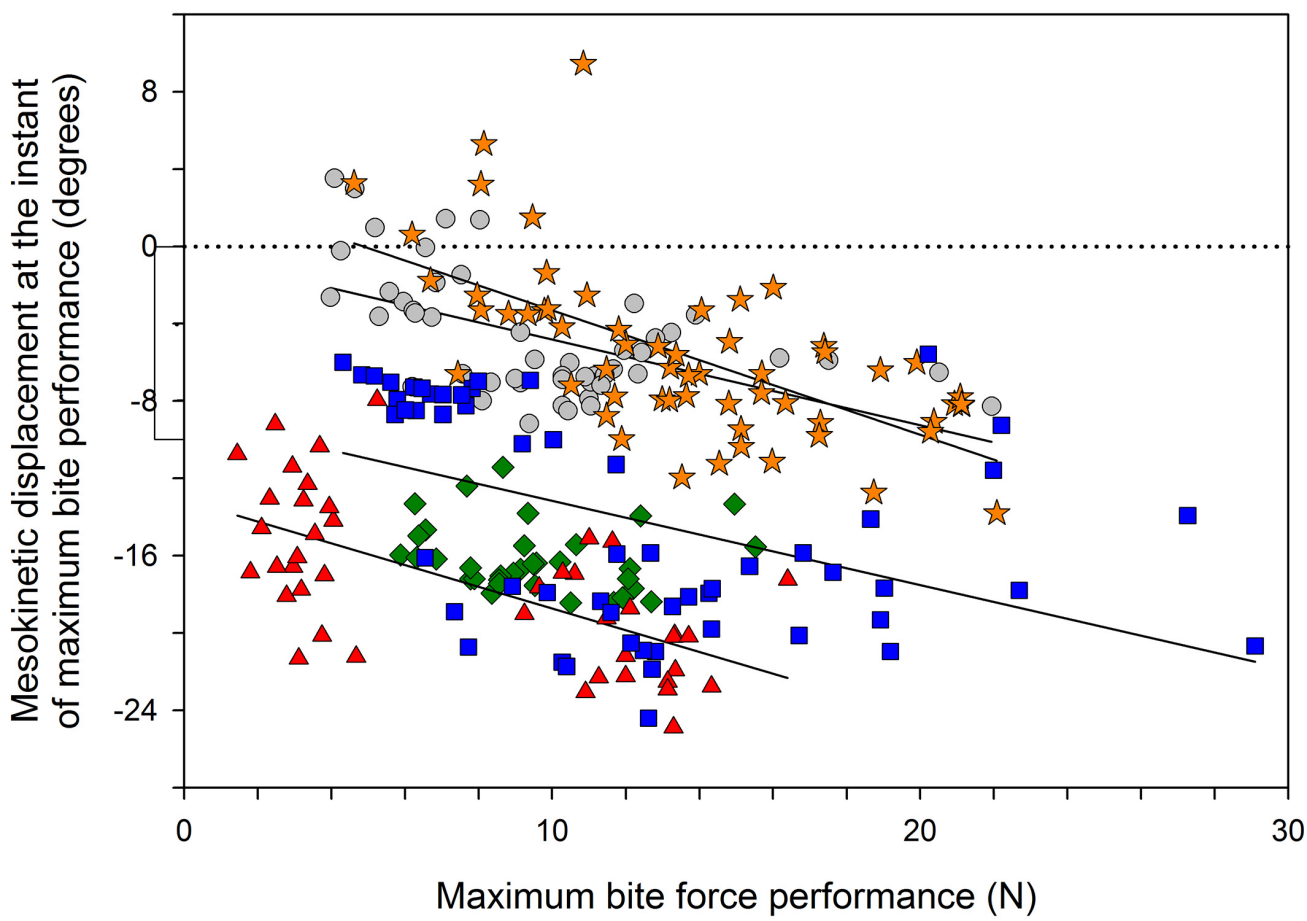
Correlation between ventral rotation of the snout relative to the braincase and maximum bite force performance in *Gekko gecko*. The correlation is significant in 4 of the 5 individuals studied (continuous lines; see Table 4 for correlation parameters), but only approaches significance across all individuals (see Table 4). Symbols represent individuals. Dotted line represents rest position: positive values indicate dorsal rotation of the snout above rest position; negative values indicate ventral rotation of the snout below rest position.

Fig 3 presents synchronized traces of bite force and mesokinetic displacement during three successive bites. Bite force recordings are synchronized with the XROMM animation so that maximum ventroflexion of the snout can be quantified for each bite separately. On average, the snout flexes ventrally 10.99° (± 0.44 s.e.m.) below its neutral position relative to the braincase during biting (Table 3). The correlation between maximum bite force and mesokinetic displacement is not significant with all individuals pooled together (Table 4). However, in four of the five individuals, bite force is significantly and positively correlated with ventral rotation of the snout relative to the braincase (Fig 7; Table 4).

### Prey transport and processing

Each feeding sequence consists of a series of jaw opening-closing cycles (i.e., gape cycles). Gape cycles were defined between two successive minimum gapes (Figs 4A and 5A). Fig 4 presents two representative feeding cycles: a puncture-crushing cycle and an intra-oral transport cycle. The puncture-crushing cycle presented is characterized by a 2.3-mm gape closing distance (Fig 4A). During the gape closing phase, the snout undergoes 3.4° of ventroflexion (i.e., total mesokinetic movement; Fig 4B), which represents a relative mesokinetic movement of -14.8° per unit of gape closing (cm^-1^). When calculating gape as if the skull were akinetic, gape would only close 0.7 mm in this cycle. Thus, ventroflexion of the snout at the mesokinetic joint increases gape closing distance by 69.6%. In contrast, during the intra-oral transport cycle presented, the gape closing distance is 6.9 mm (Fig 4A) and the snout flexes ventrally 7.8° (Fig 4B), which represents a relative mesokinetic movement of -11.3°.cm^-1^. When calculating gape as if the skull were akinetic, gape would only close 5.2 mm, indicating that mesokinetic movement provides a 24.6% increase in gape closing distance in this cycle.

Fig 5 shows a representative intra-oral transport cycle and pharyngeal packing cycle. In the intra-oral transport cycle, gape closing distance is 4.2 mm (Fig 5A) and the total mesokinetic movement is -3.5° (Fig 5B), which represents a relative mesokinetic movement of -8.33°.cm^-1^. In the akinetic model, gape would only close 2.9 mm, indicating that mesokinesis increases gape closing distance by 31.0% in this cycle. In contrast, the pharyngeal packing cycle presented is characterized by 2.1mm of gape closing distance (Fig 5A) associated with a 2.4° of ventroflexion of the snout (Fig 5B), which represents a scaled mesokinetic movement of -11.4°.cm^-1^. In the akinetic model, gape closing distance is only 1.1 mm, indicating that mesokinesis increases gape closing distance by 47.6% in this cycle.

In accordance with previous studies, gape closing distance is different among feeding cycle types. Although the cycle type x individual interaction term is significant (*F*_4,79_ = 9.20, *P* < 0.001), differences in gape closing distance between feeding cycle types are significant in each individual separately (individual 1: *F*_2,50_ = 3.52, *P* = 0.04; individual 4: *F*_2,20_ = 25.67, *P* < 0.001; individual 5: *F*_2,9_ = 20.68, *P* < 0.001). Post-hoc tests reveal that in two of the three individuals gape closing distance is significantly greater during intra-oral transport cycles than during puncture crushing cycles (*P* < 0.001 and *P* = 0.001 for individual 4 and 5, respectively) and pharyngeal packing (*P* < 0.001 and *P* = 0.004 for individual 4 and 5, respectively). No post-hoc tests are found significant in individual 1.

During jaw opening, the snout extends dorsally until approximating neutral position (Figs 4 and 5). During jaw closing, the snout flexes ventrally below its rest position in all three types of feeding cycles (Figs 4 and 5; Table 5). The cycle type x individual interaction term is significant for total mesokinetic movements (*F*_4,79_ = 6.38, *P* < 0.001), thus requiring testing for differences at the individual level. Total mesokinetic movements are significantly different among feeding cycle types in two of the three individuals (*F*_2,20_ = 21.97, *P* < 0.001 and *F*_2,9_ = 21.29, *P* < 0.001 for individual 4 and 5, respectively; *F*_2,50_ = 1.38, non-significant, for individual 1). In both of these individuals, post-hoc tests reveal that total mesokinetic movements are greater during transport cycles than during pharyngeal packing cycles (*P* < 0.001 and *P* = 0.001 for individual 4 and 5, respectively). In addition, total mesokinetic movements during puncture crushing are significantly smaller than during pharyngeal packing cycles in individual 4 (*P* = 0.003).

**Table 5:** Summary of the mesokinetic movements (mean ± standard error of the mean) observed in *Gekko gecko* during post-ingestion feeding.

Post-ingestion feeding cycle type	N	Total mesokinetic movement (degrees)^1^	Gape closingdistance (cm)	Relative mesokinetic movements(degrees / cm)^1^	Contribution of mesokinesis to gape closing distance (% of akinetic jaw closing)
Intra-oral transport	36	-7.77 ± 0.82	0.57 ± 0.06	-15.39 ± 1.17	42.03 ± 3.57
Puncture crushing	20	-4.99 ± 0.82	0.20 ± 0.02	-30.33 ± 5.23	86.27 ± 9.04
Pharyngeal packing	32	-3.07 ± 0.23	0.24 ± 0.02	-14.17 ± 1.50	60.83 ± 6.55

^1^ Negative values indicate ventral rotation of the snout during the jaw closing phase of the gape cycle.

When mesokinetic movements are scaled to gape closing distance, ANOVA reveals a significant cycle type x individual interaction term (*F*_4,79_ = 14.71, *P* < 0.001). Within individuals, significant differences in relative mesokinetic movements among cycle types are found in two of the three individuals (*F*_2,20_ = 29.76, *P* < 0.001 and *F*_2,9_ = 22.12, *P* < 0.001 for individuals 4 and 5, respectively; *F*_2,50_ = 0.55, non-significant, for individual 1). In both of these individuals, ventral flexion of the snout at the mesokinetic joint relative to gape closing distance is significantly greater during puncture crushing cycles than during intra-oral transport and pharyngeal packing cycles (post-hoc tests *P* < 0.001 for individuals 4 and 5).

Finally, simulations of the akinetic gape closing distance indicate that mesokinesis contributes to gape closing differently for each feeding cycle type (Table 5). Specifically, compared to the akinetic model, mesokinetic movements increase gape closing distance by 42% during intra-oral transport cycles, by 86% during puncture crushing cycles, and by 61% during pharyngeal packing cycles. ANOVA reveals a significant cycle type x individual interaction (*F*_4,79_ = 5.17, *P* = 0.001), and significant differences among cycle types are found in two of the three individuals (*F*_2,20_ = 17.93, *P* < 0.001 and *F*_2,9_ = 5.65, *P* = 0.03 for individual 4 and 5, respectively; *F*_2,50_ = 2.24, non-significant, for individual 1). In the significant cases, post-hoc tests reveal that the contribution of mesokinetic movement to gape closing distance is significantly greater during puncture crushing cycles than during intra-oral transport (P < 0.001 and P = 0.05 for individual 4 and 5, respectively). In addition, the contribution of mesokinetic movement during puncture crushing cycles is also significantly greater than during pharyngeal packing in individual 4 (*P* < 0.001).

## Discussion

Cranial kinesis in squamate lizards has primarily been studied by manipulating ligamentous specimens [6, 25], analyzing the morphology of intracranial sutures [7–8, 10, 20] and simulation of strain regime [18, 26–29] in a variety of squamate taxa. In contrast, experimental investigations of cranial kinesis *in vivo* are limited to only a handful of species [22–23], including two gekkotan species [21]. Consequently, the ecological relevance and biological role of cranial kinesis remain speculative and poorly understood. One hindrance to understanding the ecological and biological significance of cranial kinesis is the size of the movements that occur in freely behaving animals. Here, we show that the resolution and accuracy of XROMM is ideal for quantifying cranial kinesis in small animals (in accordance with [50]). At approximately 30 grams, *Gekko gecko* is significantly smaller than most of the other vertebrates for which XROMM data have been collected: pigs [36], ducks [17], and chukars [51]. In addition, our data are thus the first XROMM data on squamate cranial behaviors. Furthermore, even though mesokinetic movements are strictly bidimensional because of the geometry of the frontal-parietal suture (i.e., only ventro- and dorsiflexion of the snout at the mesokinetic joints are possible), the use of XROMM was essential for this study because of the considerable head and body movements that occur in 3 dimensions. As mentioned in a previous study of cranial kinesis in *G*. *gecko* [21], the utility of lateral fluoroscopy alone is limited when the subject is not restrained to the lateral plane. In this previous study, no quantitative analysis was carried out because “*G*. *gecko* (…) showed a strong tendency to tilt their heads during grasshopper feeding sequences” [21]. Using XROMM, we were not limited by these out-of-plane movements and thus were able to collect more data to complement our understanding of cranial kinesis during feeding in geckoes.

Previously, most *in vivo* data on cranial kinesis in lizards has been collected in the context of feeding [21–23]. Here, we quantified mesokinetic movements in cranial behaviors that have received less attention (but see [21], despite their definite link to survival and fitness. Indeed, gape display and defensive biting are anti-predator behaviors that are utilized by most squamate lizards, especially *G*. *gecko* [34–35]. Thus, our data expand the range of evidence for the role of mesokinesis *in vivo*. We show that the snout rotates (i) dorsally with respect to the braincase during jaw opening (Figs 2, 4 and 5), and (ii) ventrally with respect to the braincase during jaw closing (Figs 3, 4 and 5), in accordance with the four-bar linkage model of cranial kinesis [6–8]. These findings are not restricted to one particular behavior, but rather are fundamental to the three behaviors examined. Indeed, dorsiflexion of the snout occurs during gape display (Fig 2) and feeding (Figs 4 and 5) when the jaws open, and ventroflexion of the snout occurs during biting (Fig 3) and post-ingestion feeding (Figs 4 and 5) when the jaws close.

Traditionally, cranial kinesis in lizards has been proposed to have evolved with changes in the morphological constraints of the skull. In geckoes specifically, cranial kinesis has been hypothesized to be associated with bone reduction [25, 33]. The loss of the temporal bar increased the volume available for jaw adductor muscles [33, 52–53] as well as to provide mobility to the quadrate (i.e., streptostyly), as required by the four-bar linkage model. The loss of the postorbital bar has also been proposed to accommodate bigger eyes for nocturnality [25, 33]. Consequently, cranial kinesis has been considered to be “not so much functional *per se* but, instead, a consequence of the reduction of the cranial elements” [25]. Here, we demonstrate that movements at the mesokinetic joint contribute to three behaviors with significant ecological relevance.

First, *G*. *gecko* are known to use gape display behavior to establish and maintain territory against conspecifics and when threatened by a predator [34]. Our data show that mobility at the mesokinetic joint allows the snout to rotate dorsally above rest position (Fig 2) resulting in greater gapes (Fig 6; Table 4). This confirms our first hypothesis that dorsal rotation of the snout at the frontal-parietal suture contributes to an increase in gape during jaw opening. Moreover, simulating gape display in a hypothetical gekkotan skull with no mobility at the mesokinetic joint shows that mesokinesis increases maximum gape by almost 10%. This indicates that individuals with a mobile joint at the frontal-parietal suture are able to display wider gapes. If wider gape displays in *G*. *gecko* are found to be more effective in terms of survival (i.e., gape display in the context of threat response) and/or reproductive success (i.e., gape display in the context of territoriality), then the increase in maximum gape afforded by mesokinesis may be selectively advantageous. However, note that such hypothesis remains speculative at this stage and that proper experimental tests are necessary to explore the benefits of greater gape display with or without mesokinesis.

Second, when attacked by a predator, *G*. *gecko* uses bites as a defense mechanism [35]. In comparison to other lizards, bite forces in geckoes are significantly lower, and this has been proposed to be related to the presence of cranial kinesis [33]. Here, we found a positive correlation between ventroflexion of the snout and bite force in geckoes indicating that the harder the subject bites, the more ventroflexed the muzzle is (Fig 7; Table 4). This shows that mesokinetic movements in *G*. *gecko* can occur independently of jaw elevation. Indeed, during the biting experiments, all subjects maintained a firm grip on the bite force transducer–even in between bite force peaks–and thus the lower jaw could not elevate (i.e., gape distance was constant). This finding appears to contradict the 4-bar linkage model which is typically considered to be driven by the depression-elevation of the lower jaw [25]. However, this finding is in accordance with previous reports that also show that movements at intra-cranial joints are not always correlated and thus can occur independently of one another [7–8, 21, 30, 54]. Our hypothesis is that lower jaw retraction-protraction during biting may induce mesokinetic movements without jaw elevation. In this hypothesis, the retraction of the lower jaw without elevation may cause the quadrate to rotate posteriorly thereby pulling the palato-maxillary unit posteriorly. This would explain the mesokinetic movements observed here. Future XROMM investigations identifying and quantifying the different degrees of freedom of the lower jaw (i.e., both rotation and translation around the antero-posterior axis, around the medio-lateral axis, as well as around the ventro-dorsal axis) [17, 36, 50–51] would allow specific testing of this hypothesis.

Ventroflexion of the snout also occurred, as previously hypothesized [6–9], during feeding to facilitate prey processing. Thus, our study suggests that, even though cranial kinesis might have originated in the context of a reduction in cranial bones [25, 33], it also may provide organisms with significant advantages in behaviors that contribute substantially to fitness, such as feeding. Our data set is limited to post-ingestion behaviors, so the hypothesis that cranial kinesis allows alignment of the upper and lower jaws to secure prey capture remains to be tested [6–7]. Cranial kinesis in geckoes increases the velocity of jaw opening and closing during food transport and processing [33], and this may be the case during prey capture as well. This is of particular relevance since geckoes use jaw prehension to capture elusive prey. Prey capture behavior in geckoes is spontaneous and highly unpredictable, oftentimes preceded by long period of inactivity. Unfortunately, it was not possible to continually generate X-rays while waiting for the behavior. Nevertheless, based on our gape display and biting data, we would expect dorsiflexion of the snout during jaw opening and ventroflexion of the snout during jaw closing in the context of prey capture as well.

Our data are consistent with previous observations of mesokinesis in *G*. *gecko* that report 6° of flexion of the snout relative to the braincase during feeding on crickets [21]. Specifically, we show that feeding cycles involving larger gape closing movements (i.e., intra-oral transport cycles) are characterized by larger total mesokinetic movements, whereas cycles characterized by small gape closing movements (i.e., puncture crushing, pharyngeal packing) are characterized by smaller total mesokinetic movements (Table 5). Thus total mesokinetic movements are different depending on the nature of the feeding cycle (Table 5).

Although variability in gape closing movements does not diminish the insights provided by the total amount of mesokinetic movements reported here (Table 5), it may affect our understanding of the contribution of mesokinetic movements to gape closing across different types of feeding cycles. Our hypothesis was that mesokinesis is a critical component of bite force at small gapes, and regardless of the amplitude of gape closing movements, our data demonstrate that the contribution of mesokinetic movements is significantly greater during puncture crushing cycles (Table 5). Specifically, intra-oral transport and pharyngeal packing cycles are characterized by 15° of ventroflexion of the snout per unit of gape closing distance, even though the gape closing distance during intra-oral transport cycles is more than twice than during pharyngeal packing (Table 5). In contrast, even though the amplitude of gape closing movements is similar during puncture crushing and pharyngeal packing, puncture crushing cycles are characterized by 30° of ventroflexion of the snout relative to the braincase per unit of gape closing distance (Table 5). This finding is supported by the fact that the contribution of mesokinetic movements to gape closing distance is greater during puncture crushing cycles, accounting for 86% of the gape closing distance, than during intra-oral transport and pharyngeal packing cycles, accounting for 42 and 61% of the gape closing distance, respectively. In summary, intra-oral transport cycles are characterized by wide gape closing movements to which mesokinetic movements have limited contribution, whereas puncture crushing cycles are characterized by small gape closing movements achieved by proportionally larger movements of the snout at the mesokinetic joint. Finally, pharyngeal packing cycles are characterized by small gape closing movements with little contribution from mesokinetic movements. Given that the diet of geckoes is composed primarily of arthropods with a hard cuticle [47], cranial kinesis may enhance the ability to pierce the protective cuticle of the prey, especially since bite force in geckoes is relatively low in comparison to other lizards of similar size [33]. This is particularly relevant when feeding on active prey like arthropods because it allows efficient food processing while maintaining a firm grasp of the prey, thus reducing the chances of prey escape. As cranial kinesis also increases the velocity of jaw opening and closing movements [33], organisms feeding on elusive prey may benefit from the functional advantages of cranial kinesis.

In another gekkotan species, *Phelsuma madagascariensis*, mesokinetic movements during feeding on grasshoppers are greater than in *G*. *gecko* feeding on crickets. This may reflect interspecific differences in the range of movements at intracranial joints [21], the effects of prey size or material properties, or both. Teasing apart the basis of these differences requires more controlled feeding experiments in both species using foods spanning a range of sizes and material properties. These data are essential for understanding how mesokinesis impacts flexibility of feeding behavior (*sensu* [55]) and the potential role of cranial kinesis in the evolution of dietary specialization. Previous study of cranial kinesis in a different lizard, *Urimastyx acanthinurus*, feeding on locusts also report that movements at the streptostylic joint differ significantly between intra-oral transport and swallowing cycles [56]. However, no differences were found between cycle types when the subject was eating on immobile food item (i.e., endive), thus emphasizing the importance of prey mobility in understanding cranial kinesis.

**Fig 8:**
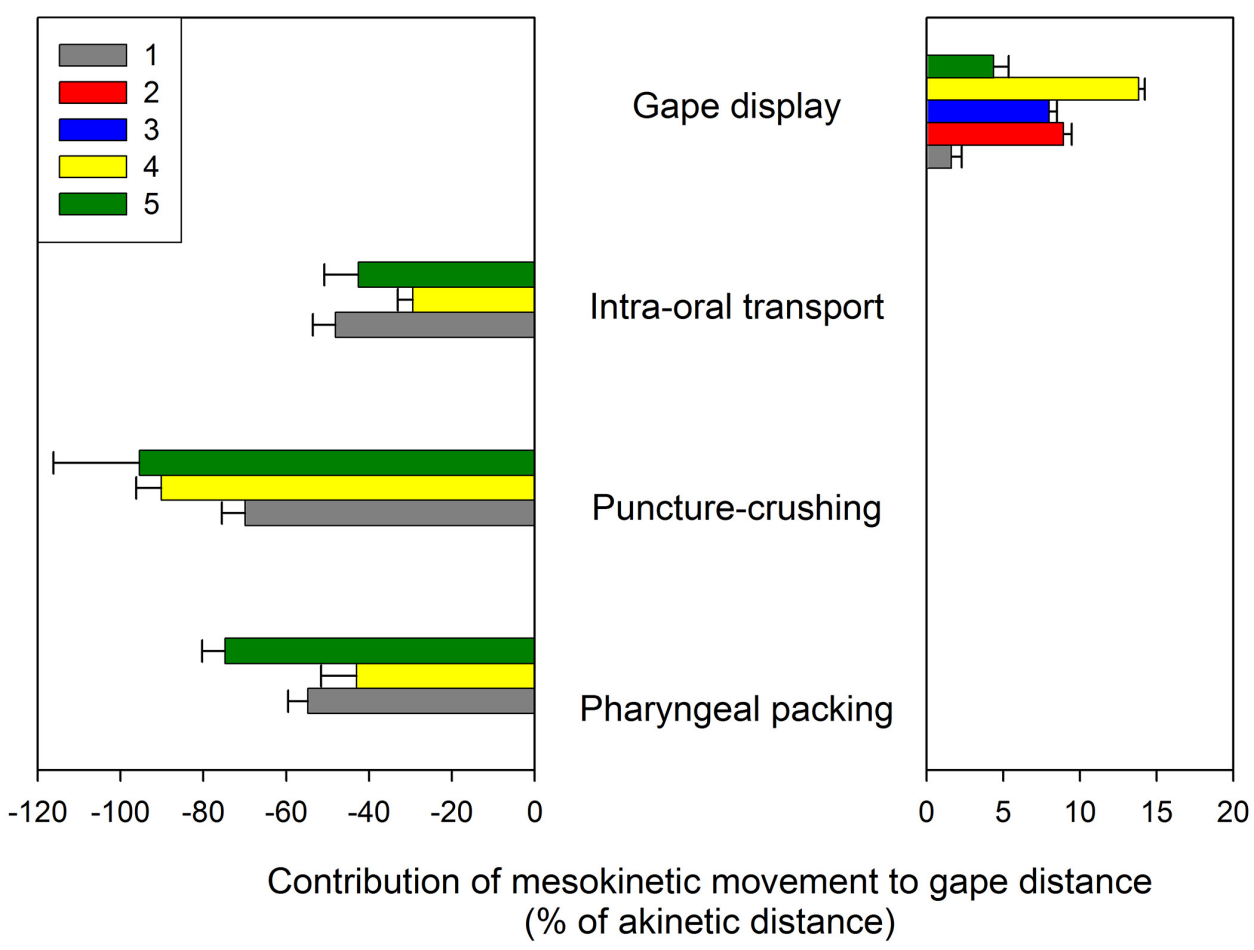
Summary of the contribution of mesokinetic movement to jaw gape distance in *Gekko gecko* during gape display, intra-oral food transport and processing behaviors in the 5 individuals studied. Positive values indicate that dorsal rotation of the snout above rest position increases gape opening distance in comparison to a theoretical akinetic skull where gape distance is solely induced by the depression of the lower jaw. Negative values indicate that ventral rotation of the snout below rest position increases gape closing distance without lower jaw elevation. Colors represent individuals, note that no feeding data could be collected for individuals 2 (red) and 3 (blue).

For all individuals, maximum dorsiflexion of the snout coincided with maximum gape and maximum ventroflexion of the snout coincided with maximum bite force. Nevertheless, we found differences between subjects in the correlations between the magnitudes of these variables, as well as in the overall contribution of mesokinesis to jaw movements (Fig 8). First, maximum gape in one individual was not significantly correlated with maximum dorsiflexion of the snout (grey circles in Fig 6; see also Table 4). This individual displayed the smallest gape, which may indicate that it performed submaximally across the study. Second, maximum bite force in another individual was not significantly correlated with ventroflexion of the snout (green diamonds in Fig 7; see also Table 4). This individual used prolonged bites so that the bite force recordings had a plateau followed by a slow decline in force, rather than an instantaneous bite force peak as in the other individuals. Because of this, we evaluated the first instant of maximum bite force in this individual. Given these differences, it is possible that mesokinesis facilitates rapid and short bites, much like puncture crushing, rather than prolonged biting. Despite no correlation between mesokinesis and bite force in this one individual, its snout did rotate ventrally below rest position during puncture crushing, confirming the presence of mesokinesis during feeding.

While we have proposed behavioral explanations for the inter-individual differences observed in our study, individual differences in cranial kinesis have been reported previously in squamate taxa [6–8, 20]. However, the basis for these differences is not well understood. Age may be a factor if changes in joint structure occur with changes in body size, e.g., joint fusion. The subjects used in our study were purchased through the same commercial dealer and it is thus hard to quantify their individual life histories. However, we do not feel this was a complicating factor here as all subjects were of similar size suggesting a similar age. Moreover, as the overall trends across all individuals demonstrate that mesokinetic movements (i) increase maximum gape during gape display (Fig 6), (ii) are positively correlated with bite force (Fig 7), and (iii) contribute significantly to prey crushing during feeding (Table 5), our study suggests that mesokinesis may be subjected to selective pressure. To this end, future *in vivo* investigations on the role of other types of cranial kinesis in facilitating cranial behaviors will further our understanding of the evolution of the vertebrate cranial system.

## Supporting Information

S1 File *X-ray Reconstruction Of Moving Morphology (XROMM) of feeding behavior in Geckoes*. The video shows 2 consecutive gape cycles: an intra-oral transport cycle and a puncture-crushing cycle. Mesokinesis, i.e., movements of the snout (yellow) relative to the braincase (red) at the frontal-parietal suture is a key component of gape closing movements.
(PDF)

S2 File *Raw data file compiling the data used in the analyses*. Gape display data are: maximum gape distance (cm), mesokinetic displacement (degrees) and the contribution of mesokinetic displacement (% of akinetic gape distance). Bite data are: bite force (N) and mesokinetic displacement (degrees). Feeding data are: gape closing distance (cm), mesokinetic displacement (degrees) and the contribution of mesokinetic displacement (% of akinetic gape distance). The different feeding behaviors are coded with different numbers. Data are presented for all sequences recorded for each individuals.
(XLSX)

S3 File Completed “ARRIVE Guidelines Checklist” for reporting animal data in this manuscript. (PDF)
