# Supplementary material for: In Vivo Measurement of Mesokinesis in Gekko gecko: The Role of Cranial Kinesis during Gape Display, Feeding and Biting
Source: PLoS One. 2015 Jul 31;10(7):e0134710. doi: 10.1371/journal.pone.0134710 (PMC4521707; doi:10.1371/journal.pone.0134710)

Montuelle SJ & Williams SH – ***In vivo* measurement of mesokinesis in *Gekko gecko*: the role of cranial kinesis during gape display, feeding and biting.**

<u>**SUPPORTING INFORMATION S1**</u>. ***X-ray Reconstruction Of Moving Morphology (XROMM) of feeding behavior in Geckoes.*** The video shows 2 consecutive gape cycles: an intra-oral transport cycle and a puncture-crushing cycle. Mesokinesis, i.e., movements of the snout (yellow) relative to the braincase (red) at the frontal-parietal suture is a key component of gape closing movements.

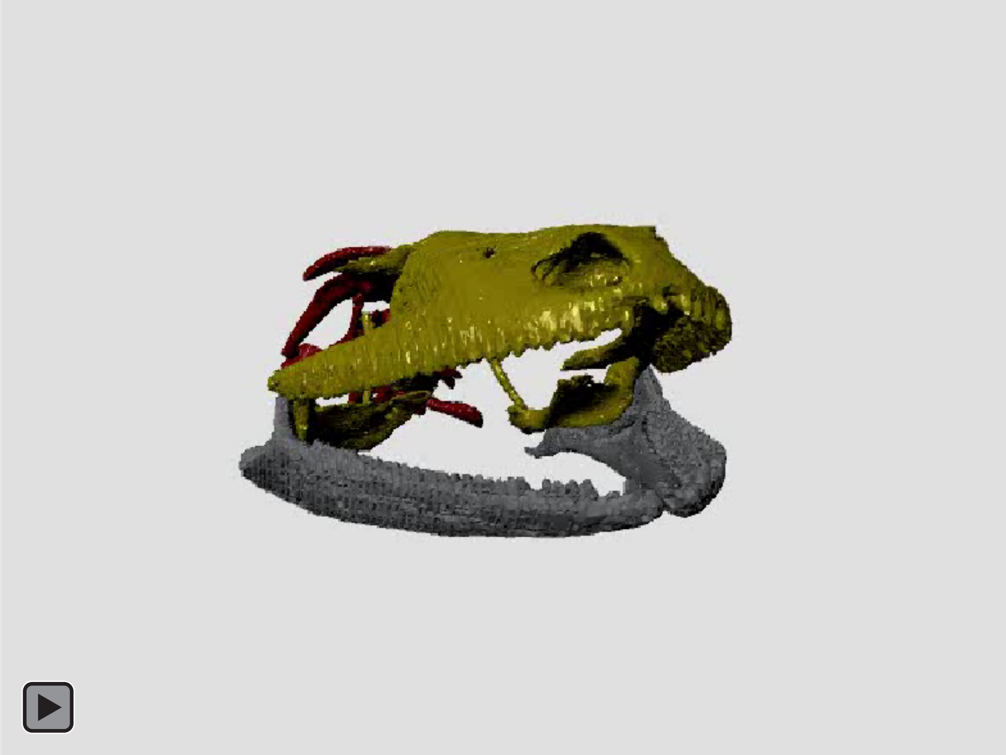

Supplement: S1 File — The video shows 2 consecutive gape cycles: an intra-oral transport cycle and a puncture-crushing cycle. Mesokinesis, i.e., movements of the snout (yellow) relative to the braincase (red) at the frontal-parietal suture is a key component of gape closing movements. (PDF) [file pone.0134710.s001.pdf]
